# Highlighting the Importance of Signaling Pathways and Immunohistochemistry Features in HCC: A Case Report and Literature Review

**DOI:** 10.3390/reports8040197

**Published:** 2025-10-03

**Authors:** Alexandru Madalin Hasan, Ioana Larisa Paul, Simona Cavalu, Ovidiu Laurean Pop, Lorena Paduraru, Ioan Magyar, Mihaela Doina Chirila

**Affiliations:** Faculty of Medicine and Pharmacy, University of Oradea, P-ta 1 Decembrie 10, 410087 Oradea, Romania; alexhasy@yahoo.com

**Keywords:** hepatocellular carcinoma, PYGO2, PIK3CA, hTERT, MAPK

## Abstract

**Background and Clinical Significance:** In hepatocellular carcinoma (HCC), numerous signaling pathways become aberrantly regulated, resulting in sustained cellular proliferation and enhanced metastatic potential. Tumors that lack PYGO2 may not show the same types of tissue remodeling or regenerative features driven by the Wnt/β-catenin pathway, which could make the tumor behave differently from others that are Wnt-positive. PIK3CA-positive tumors are often associated with worse prognosis due to the aggressive nature of the PI3K/AKT pathway activation. This is linked to higher chances of metastasis, recurrence, and resistance to therapies that do not target this pathway. **Case presentation:** In this paper we present a rare case of hepatocellular carcinoma with PIK3CA-positive and PYGO2-negative signaling pathways, several key aspects of the tumor’s behavior, prognosis, and treatment options. Although alpha-fetoprotein (AFP) levels were significantly elevated, the CT and MRI examination showed characteristics of malignancy, HCC with secondary hepatic lesions and associated perfusion disturbances. The case particularities and immunohistochemistry features are highlighted in the context of literature review, the PIK3CA mutation suggesting the activation of the PI3K/AKT/mTOR pathway, a critical signaling pathway involved in cell survival, proliferation, and metabolism. **Conclusions:** Due to the aggressive nature of PIK3CA mutations, close monitoring and consideration of immunotherapy and targeted treatments are of crucial importance.

## 1. Introduction and Clinical Significance

Several signaling pathways are deregulated in HCC and contribute to uncontrolled proliferation and metastasis. Targeting these pathways, such as PI3K/AKT/mTOR and Wnt/β-catenin, has revealed novel treatment options [1,2]. However, the physiological roles of these pathways in normal hepatocytes must be considered to distinguish between oncogenic activation and homeostatic regulation. In healthy liver tissue, the PI3K/AKT/mTOR pathway modulates metabolic activity and cell survival, while the Wnt/β-catenin pathway plays essential roles in hepatocyte renewal and regeneration. Their aberrant activation in HCC leads to aggressive tumor behavior, therapeutic resistance, and poor prognosis [1,2,3].

In the context of drug development, two signaling axes have predominantly underpinned modern therapeutic strategies in HCC: the PI3K/AKT/mTOR pathway—targeted by mTOR/PI3K inhibitors or rational combinations—and the Wnt/β-catenin pathway, which shapes tumor biology and immune exclusion and is being explored for indirect therapeutic modulation. In parallel, immune checkpoint blockade has transformed first-line systemic therapy (e.g., atezolizumab–bevacizumab and the STRIDE regimen), and is often rationalized by pathway biology and the tumor–immune microenvironment.

In hepatocellular carcinoma (HCC), numerous signaling pathways become aberrantly regulated, resulting in sustained cellular proliferation and enhanced metastatic potential. Therapeutic strategies aimed at selectively modulating these dysregulated pathways—particularly those implicated in key oncogenic processes such as uncontrolled cell division, migration, and dissemination—hold promise in mitigating disease progression. Notably, targeting pivotal signaling networks, including receptor tyrosine kinase pathways, the Ras/Raf/mitogen-activated protein kinase (MAPK) cascade, the phosphoinositide 3-kinase (PI3K)/AKT/mammalian target of rapamycin (mTOR) axis, Wnt/β-catenin signaling, the ubiquitin–proteasome degradation system, Hedgehog signaling, as well as pathways involving hTERT, PIK3CA, PYGO2, and NTRK, has facilitated the identification of novel therapeutic agents for the clinical management of HCC [3,4].

PYGO2, a mammalian homolog of the *Drosophila Pygopus* protein, plays a critical role in early embryonic development and is involved in the morphogenesis of multiple tissues, including the lens, brain, lung, kidney, and hair follicles. Additionally, it contributes to the regenerative proliferation of the skin. The PYGO protein family was initially identified in *Drosophila melanogaster* and *Xenopus*, with two homologs recognized in mammals—PYGO1 and PYGO2. Among these, PYGO2 is considered more functionally significant due to its higher expression levels relative to PYGO1. Notably, aberrant overexpression of PYGO2 has been observed in various malignancies, including hepatocellular carcinoma, as well as ovarian, breast, cervical, and lung cancers [5].

PIK3CA mutations or amplifications are commonly observed in HCC, leading to constitutive activation of the pathway. Altered regulation of upstream RTKs (e.g., EGFR, FGFR) or mutations in negative regulators like PTEN (phosphatase and tensin homolog) can also enhance PI3K pathway activity. Constitutive AKT activation results in increased cell proliferation, resistance to apoptosis, enhanced angiogenesis, and metabolic reprogramming [6]. The PI3K/AKT pathway interacts with other pathways frequently dysregulated in HCC, such as the Wnt/β-catenin pathway, Ras/MAPK pathway, and JAK/STAT pathway. Such interactions amplify oncogenic signals and contribute to resistance to targeted therapies [7,8]. Canonical hotspot mutations occur in exon 9 (E542K, E545K) and exon 20 (H1047R/H1047L), which increase p110α kinase activity; however, their frequency in HCC varies by cohort and is generally low compared with other epithelial cancers.

In this case report we identified and interpreted the role of PIK3CA-positive and PYGO2-negative signaling pathways in the development and initiation of HCC, highlighting the histopathological and immunohistochemical features, in the context of a literature review. To our knowledge, the immunophenotype combining PIK3CA-positive and PYGO2-negative staining in HCC is rarely documented; we therefore report this case and discuss its implications for tumor biology and therapy.

## 2. Case Presentation

### 2.1. Clinical Features

Key admission laboratory abnormalities (Table 1) included marked anemia (HGB 7.6 g/dL), thrombocytopenia (80 × 10^9^/L), coagulopathy (INR 3.01; prolonged PT/APTT), cholestatic and cytolytic liver injury (AST 125 U/L, ALT 180 U/L, ALP 289 IU/L, total bilirubin 3.2 mg/dL), hypoalbuminemia (2.2 g/dL), elevated LDH (360 U/L), systemic inflammation (CRP 52 mg/dL; ESR 50 mm/h), and a markedly elevated AFP (420 ng/dL). Viral hepatitis markers were negative. These findings, together with the imaging pattern, supported the diagnosis of HCC with impaired hepatic reserve.

A 55-year-old woman was admitted to the emergency department for acute pain in the right upper abdominal quadrant, pain in the right shoulder, weakness, and tiredness; the patient related loss of her appetite too. Physical examination revealed scleral and skin jaundice, mild hepatomegaly, bloating in the gastric area, but no splenomegaly or ascites. The BMI was 33 kg/m^2^, and laboratory blood tests conducted in the emergency department suggested liver dysfunction. The AFP levels were elevated (420 ng/dL), while the imaging investigation (CT and MRI) demonstrated classic features of HCC, including arterial phase hyperenhancement and venous washout. The diagnosis was further confirmed through histopathological evaluation and immunohistochemical analysis.

To complete the investigations, the patient was admitted to the Gastroenterology department, Emergency Clinical Hospital Oradea, Bihor County, Romania. Additionally, the patient’s medical history included diabetes mellitus type II, which was under medication with Metformin 1000 mg/day and a moderate hypertension (second stage) treated with Ramipril 5 mg and Amlodipine 10 mg. A family history revealed the following: mother—hypertension and colon cancer; father—COPD; sister—diabetes mellitus type II and amyloidosis. Table 1 displays the blood test results, showing slight neutrophilia, moderate hypochromic, microcytic anemia, hepatic cytolysis syndrome, hypoalbuminemia a, high bilirubin levels, coagulopathy, and a high rate of proliferation due to an increased LDH, while viral markers were negative.

### 2.2. Imaging (CT and MRI)

**Figure 1:**
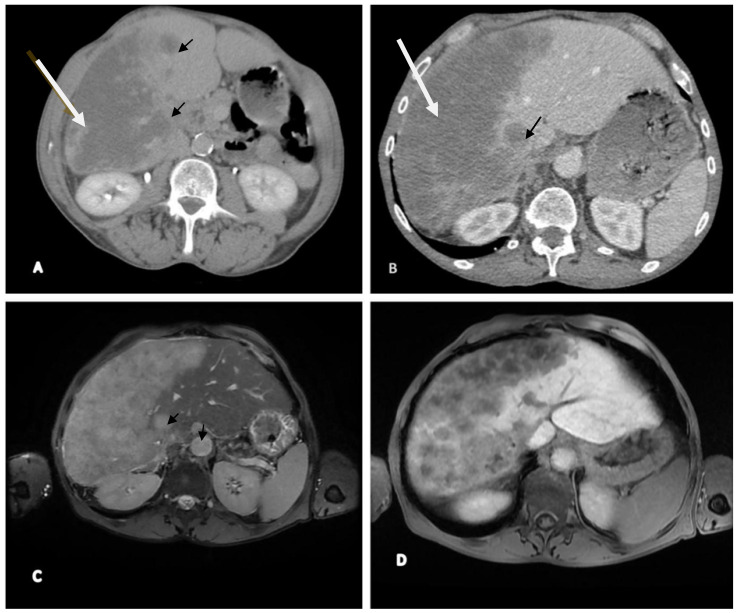
CT (**A**,**B**) and MRI (**C**,**D**) examination. Contrast-enhanced axial CT images of the liver acquired at multiple phases demonstrate a large, well-defined enhancing mass located in the right hepatic lobe (indicated by the large arrow), consistent with primary hepatocellular carcinoma (HCC). Additionally, a smaller enhancing lesion is observed in the left hepatic lobe (indicated by the small arrow), suggestive of intrahepatic metastasis. (**A**) On the non-contrast (plain) scan, both lesions present as hypodense relative to the surrounding liver parenchyma. (**B**) During the arterial phase, both lesions exhibit early arterial enhancement characteristic of HCC. Liver enlarged, heterogeneous structure, irregular contour, lobulated at the level of the left lobe, almost entirely occupied by multiple nodular lesions, both isolated and well-defined, with axial diameters up to approximately 3.3 cm (**C**). In T2 hyperintensity and T1 hypointensity, with moderate diffusion restriction in DWI/ADC sequences, post-contrast enhancement shows hypointense lesions with multiple non-enhancing areas inside (most likely necrosis), and areas that compress the right portal branches, which appear filiform and homogeneously opacified post-contrast. Lesions exhibit hypointensity compared to the surrounding parenchyma, with characteristics of malignancy—HCC with secondary hepatic lesions and associated perfusion disturbances (**D**).

Our medical investigation continued with the CT scan liver evaluation and MRI; the procedure was completed by administrating a radiocontrast agent (Primovist on standard dose of 0.1 mL/kg), the details being presented in Figure 1A,B. Upon CT examination, the imaging findings were highly suspected for malignancy, so we further performed an MRI to establish the diagnosis (Figure 1C,D).

### 2.3. Histopathological and Immunohistochemistry Analysis

**Figure 2:**
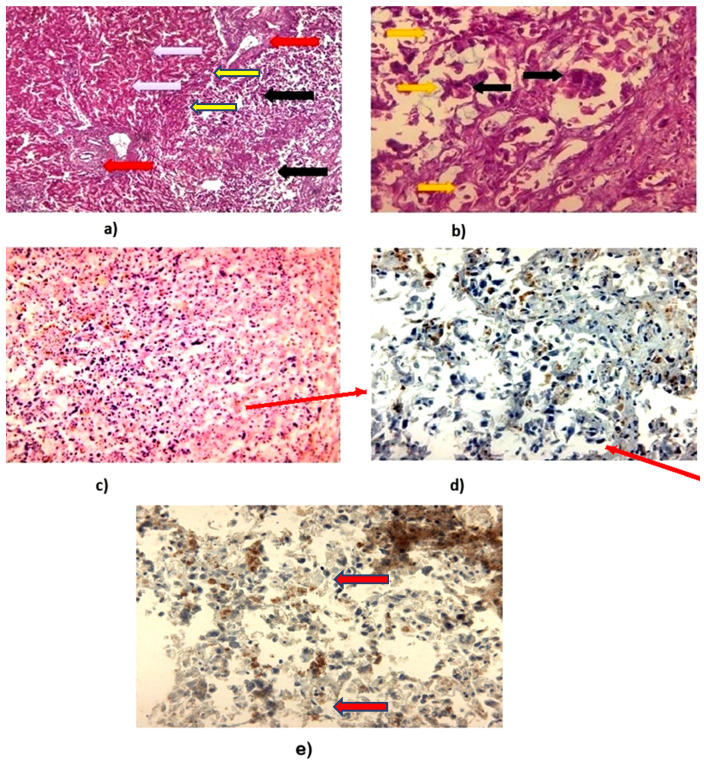
(**a**) Normal liver parenchyma (yellow arrow) with presence of portal spaces (red arrow). The right side of the image shows a change in the normal histological architecture by a tumor-HCC proliferation (black arrow), 40× H&E. (**b**) Hepatocarcinoma tumor proliferation. The image identifies a cellular proliferation with a pseudoglandular arrangement. Cells with pronounced pleomorphism in size and shape are noted. At high magnitude, tumor cells of different sizes (yellow arrow) and anisokaryosis (black arrow) are identified, 400× H&E. (**c**) Hematoxylin and eosin (H&E) staining revealed hepatic tissue architecture with malignant transformation. The positive control was performed on normal liver tissue, while the negative control was obtained by omitting the staining reagent, 40×. (**d**) The immunohistochemical reaction is negative for PYGO2. The positive control was performed on nerve tissue and the negative control by omitting the antibody, 40×. (**e**) The immunohistochemical reaction is positive for PIK3CA. Granular cytoplasmic labeling is seen in most tumor cells (red arrows). In some carcinoma cells, perinuclear labelling is enhanced in the context of activation of the intracellular mechanism, 40×.

A biopsy of the hepatic lesion was procured and subjected to histopathological evaluation. As shown in Figure 2, routine hematoxylin and eosin (H&E) staining of formalin-fixed, paraffin-embedded tissue sections—visualized at increasing magnifications—reveals a diffuse distribution of malignant cells throughout the specimen. Immunohistochemical analysis highlights the expression patterns of PYGO2 and PIK3CA. At high magnification, malignant cells are observed infiltrating the stromal tissue, characterized by prominent nuclear staining, pleomorphic nuclei, and the presence of mitotic figures, indicative of active cellular proliferation. No significant fibrosis, MASLD, or chronic viral hepatitis was identified.

## 3. Discussion

Primary liver cancer ranks as the seventh most commonly diagnosed cancer worldwide and represents the second leading cause of cancer-related mortality. Hepatocellular carcinoma (HCC) constitutes approximately 75% of all primary liver cancer cases globally, making it the predominant histological subtype. In Romania, recent epidemiological data report an incidence ranging from 4 to 10 cases per 100,000 individuals annually [9]. The pathogenesis and progression of HCC follow a multistep process, involving a transition from hyperplastic to dysplastic nodules, and subsequently to early and advanced stages of carcinoma. This progression is driven by imbalances between apoptosis and cellular proliferation. Numerous studies have identified alterations in gene expression profiles, chromosomal amplifications, mutations, deletions, copy number variations, somatic mutations, CpG island hypermethylation, and global DNA hypomethylation—molecular events that may serve as potential therapeutic targets [10]. Notably, activation of Wnt/β-catenin (CTNNB1) defines an immune-excluded HCC subclass and has been linked to reduced benefit from PD-1/PD-L1 blockade.

The initiation of proliferative signaling cascades is commonly mediated by the binding of growth factors to their respective receptor tyrosine kinases, resulting in the activation of protein-phosphorylating enzymes that relay signals to the nucleus. Key growth factors such as epidermal growth factor (EGF), transforming growth factors α and β (TGF-α/β), insulin-like growth factor (IGF), and vascular endothelial growth factor (VEGF) play crucial roles not only in oncogenesis but also in physiological liver regeneration following injury. Moreover, members of the fibroblast growth factor (FGF) and platelet-derived growth factor (PDGF) families are implicated in both hepatic fibrosis and HCC progression [11]. Among the most critical signaling pathways implicated in HCC pathophysiology are hTERT, PIK3CA, PYGO2, and NTRK, which represent promising targets for molecularly guided therapeutic interventions.

A thorough review and synthesis of previously published studies provided a comprehensive overview of the predominant mutations associated with the PYGO2 and PIK3CA signaling pathways. In the present case, characterized by a rare HCC profile with PIK3CA positivity and PYGO2 negativity, the molecular profile suggests an aggressive tumor phenotype driven by activation of the PI3K signaling axis. The patient experienced rapid clinical deterioration, requiring hospitalization for hepatic dysfunction and systemic symptoms, as detailed in the laboratory data and imaging sections. The background liver condition also warrants further consideration. Although no significant fibrosis, MASLD, or chronic viral hepatitis was identified, subtle liver injury cannot be excluded. Assessing the liver microenvironment in relation to molecular signatures may provide deeper insights into HCC pathogenesis and treatment responsiveness.

Therapeutic strategies in such cases are expected to prioritize inhibition of the PI3K/AKT/mTOR pathway. Conversely, the absence of PYGO2 expression indicates that targeting the Wnt/β-catenin pathway may have limited clinical benefit. Given the established association between PIK3CA mutations and tumor aggressiveness, rigorous clinical surveillance, along with the exploration of targeted therapies and potential immunotherapeutic approaches, is strongly warranted. Previous studies associate PYGO2 with tumor stemness and invasiveness, yet our findings point to the possibility that PYGO2-negativity does not preclude aggressive behavior, especially in the presence of PI3K pathway activation. The contribution of each pathway should be considered in both tumor and non-tumoral liver tissue to fully understand the signaling imbalance. Future studies should aim to include matched normal liver samples to validate molecular changes and their impact on HCC biology [12].

### 3.1. PIK3CA Mutations and Their Role in Hepatocellular Carcinoma

Regarding immunotherapy resistance, PYGO2 functions as a chromatin-bound co-activator that stabilizes β-catenin-dependent transcription. Although direct clinical data in HCC are limited, preclinical and translational studies indicate that β-catenin activation is associated with T-cell exclusion and primary resistance to PD-1/PD-L1 inhibitors; therefore, PYGO2 loss could theoretically mitigate Wnt signaling output, while PYGO2 overexpression may contribute to immune evasion.

PIK3CA mutations are observed in a substantial subset of hepatocellular carcinoma (HCC) cases and are recognized as key drivers of tumor initiation and progression. These genetic alterations result in constitutive activation of the phosphoinositide 3-kinase (PI3K)/Akt signaling pathway, which plays a pivotal role in promoting cell survival, resistance to apoptosis, and uncontrolled cellular proliferation. Specifically, mutations in PIK3CA enhance the kinase activity of PI3K, thereby contributing to aberrant cell growth and malignant transformation [13]. Activation of the PI3K/Akt/mammalian target of rapamycin (mTOR) signaling axis also facilitates angiogenesis—promoting the formation of new blood vessels—which enhances tumor vascularization and supports tumor progression. Furthermore, this pathway regulates epithelial–mesenchymal transition (EMT), a biological process that endows cancer cells with increased motility and invasiveness, thereby promoting metastatic dissemination to distant organs [14].While PIK3CA mutations are important in certain HCC subtypes, a significant proportion of HCC cases are associated with chronic viral infections, particularly hepatitis B virus (HBV) and hepatitis C virus (HCV) [15].These infections induce persistent hepatic inflammation and tissue injury, contributing to carcinogenesis through mechanisms distinct from PI3K pathway activation. In virally induced HCC, oncogenic signaling is often mediated by inflammatory pathways, including nuclear factor-kappa B (NF-κB) and signal transducer and activator of transcription 3 (STAT3), both of which are implicated in immune-mediated inflammation and fibrotic remodeling of liver tissue [16]. In this inflammatory context, PIK3CA mutations occur less frequently compared to other molecular alterations, such as *TP53* loss-of-function mutations or downregulation of microRNA-122 (MIR-122). These alternative pathways underscore the heterogeneity of HCC pathogenesis and highlight the importance of molecular profiling for personalized therapeutic approaches [17].

Table 2 summarizes the most frequently reported PIK3CA mutations, their mechanisms of action, and associated cancer types, as documented in the current literature.

Hotspot Mutations: These mutations, particularly in Exon 9 and Exon 20, are the most prevalent and have a significant impact on PI3K pathway activation, making them central targets for therapeutic approaches like PI3K inhibitors.

Point Mutations (Missense): Often result in single amino acid changes that can affect protein function and kinase activity, playing a key role in various cancers.

In-frame Insertions/Deletions (Indels): Less frequent but still important, these mutations can also drive aberrant signaling by disrupting the normal structure of the protein.

Copy Number Variations (CNVs): Gene amplifications often lead to overexpression of PIK3CA, which results in enhanced PI3K signaling and tumorigenesis, providing potential targets for therapies aimed at reducing gene expression.

Complex Mutations and Fusions: While rare, these mutations may create new forms of oncogenic PIK3CA or involve interaction with other genes, influencing treatment strategies. Loss-of-function Mutations: These are more commonly observed in benign or non-cancerous conditions, and in rare cases, they can influence tumor suppression.

### 3.2. PYGO2 Mutations

Recent studies have identified PYGO2 as a critical co-activator of the Wnt/β-catenin signaling pathway. Given the well-established role of Wnt/β-catenin dysregulation in the development and progression of various malignancies, it is reasonable to infer that PYGO2 also plays a significant role in tumorigenesis and cancer progression [24].

In hepatocellular carcinoma (HCC), elevated PYGO2 expression has been positively correlated with increased tumor size, vascular invasion, and poor tumor differentiation. Ephrin A4 (EFNA4), a molecule known to act as an oncogene in HCC, has been shown to enhance HCC cell proliferation by interacting with and upregulating PYGO2. A study by Weidong et al. demonstrated that silencing EFNA4 led to a reduction in cell proliferation, invasion, angiogenesis, and Wnt/β-catenin signaling by downregulating PYGO2 expression [25]. Functionally, PYGO2 contains two essential domains: the NHD (N-terminal homology domain), which modulates transcriptional activation, and the PHD (plant homeodomain), which mediates interaction with the N-terminal region of β-catenin via adaptor proteins [26]. Multiple studies have confirmed that PYGO2 is highly expressed at both mRNA and protein levels in HCC tissues, implicating its role in tumor progression, particularly through the promotion of cell migration. This pro-migratory effect may be partially explained by PYGO2’s regulatory influence on the CDH1 (E-cadherin) promoter. PYGO2 binding to this promoter represses E-cadherin expression, a key molecule involved in cell–cell adhesion. Zhang et al. demonstrated that knockdown of PYGO2 results in increased E-cadherin expression, leading to enhanced cellular adhesion and reduced invasive potential. Consequently, diminished PYGO2 levels correlate with decreased metastatic capability.

Moreover, PYGO2 is implicated in chromatin remodeling through its ability to bind methylated lysine 4 residues on histone H3 (H3K4me), a marker of active transcription. It has also been shown to promote trimethylation of H3K4 and acetylation of histone H3 at lysine9 and 14 (H3K9/K14), both being associated with transcriptional activation [27].

In addition to its chromatin-related functions, PYGO2 appears to act as a scaffold protein, facilitating interactions between β-catenin (CTNNB1), HNMT, TMPRSS11D (a histone acetyltransferase), and chromatin components. This scaffolding role enhances signal transduction via the Wnt pathway and contributes to the nuclear retention of β-catenin, further amplifying transcriptional responses associated with oncogenic transformation [28].

The role of PYGO2 in hepatocellular carcinoma (HCC) is an emerging area of research, particularly due to its involvement in glycosylphosphatidylinositol (GPI) anchor biosynthesis. GPI anchors are crucial for anchoring certain proteins to the cell membrane, affecting their function in cell signaling, adhesion, and immune response. When PYGO2 is negative in HCC, it suggests that the gene may not be expressed or that the protein it encodes is not functioning as expected in the tumor cells. This could have several potential implications: Disrupted GPI Anchor Biosynthesis: The PYGO2 gene is involved in the biosynthesis of GPI anchors, which are essential for attaching proteins to the cell membrane. Loss of this function may affect the membrane localization of important tumor suppressor proteins or other regulatory molecules, potentially contributing to tumorigenesis.

Immune Evasion: GPI-anchored proteins are often involved in immune cell signaling. If PYGO2 is downregulated or absent, HCC cells may alter their immune profile, which could lead to immune evasion or altered responses to immune therapies [29]. Prognostic Implications: The absence of PYGO2 could serve as a prognostic marker, suggesting that the tumor may be more aggressive or resistant to certain treatments, depending on how the loss of PYGO2 impacts the cell’s characteristics. Molecular Pathways: The loss of PYGO2 may influence various signaling pathways involved in cell adhesion, migration, and survival, which are critical in cancer progression and metastasis. Table 3 provides a more detailed look at how mutations in PYGO2 contribute to cancer pathology, highlighting its potential role in initiating and progressing various cancers by disrupting key cellular processes.

Wnt/β-catenin Pathway Activation: Mutations in PYGO2 often lead to dysregulation of the Wnt signaling pathway. Overactive Wnt signaling promotes cell proliferation, migration, invasion, and stemness, which are hallmarks of cancer progression [37].

Tumor Suppression Loss: PYGO2 mutations that lead to the loss of function may impair tumor suppressor pathways, making cells resistant to apoptosis and increasing their proliferative potential.

Metastatic Potential: Overexpression of PYGO2 or dysregulation of its regulatory regions can promote epithelial-to-mesenchymal transition (EMT), facilitating tumor metastasis and dissemination to distant organs.

The PIK3CA gene encodes the p110α catalytic subunit of phosphoinositide 3-kinase (PI3K), a central regulator of the PI3K/AKT/mTOR signaling cascade. This pathway plays a critical role in cellular processes such as proliferation, survival, metabolism, and angiogenesis. Activation of PI3K occurs in response to extracellular stimuli, typically via receptor tyrosine kinases (RTKs)orG-protein-coupled receptors (GPCRs), upon binding of growth factors such as epidermal growth factor (EGF) or insulin-like growth factor 1 (IGF-1). Upon activation, PI3K phosphorylates phosphatidylinositol-4,5-bisphosphate (PIP2) to generate phosphatidylinositol-3,4,5-trisphosphate (PIP3) [37,38]. PIP3 acts as a second messenger by recruiting AKT (Protein Kinase B) to the plasma membrane, where it undergoes phosphorylation and full activation through PDK1 and mTORC2. Activated AKT subsequently initiates a series of downstream signaling events that drive oncogenic processes.

#### Oncogenic Effects of PI3K/AKT/mTOR Activation in Hepatocellular Carcinoma (HCC)

Cell Proliferation: AKT stimulates mTORC1, a major promoter of protein synthesis and cell growth, thus enhancing cellular proliferation.

Cell Survival: AKT inactivates pro-apoptotic proteins such as BAD, while simultaneously activating anti-apoptotic factors, contributing to resistance against chemotherapy and radiotherapy.

Angiogenesis: The PI3K/AKT pathway upregulates the expression of vascular endothelial growth factor (VEGF), promoting angiogenesis and enhancing tumor vascularization.

Metastasis: Activation of this signaling axis supports epithelial–mesenchymal transition (EMT), a key step in tumor cell invasion and metastasis. In hepatocellular carcinoma, aberrant activation of the PI3K/AKT/mTOR pathway—often due to PIK3CA mutations—is closely associated with aggressive tumor behavior and poor clinical outcomes. These insights underscore the potential of this pathway as a therapeutic target for molecular intervention [37,38].

### 3.3. Challenges and Treatment Management

The treatment of hepatocellular carcinoma (HCC) is particularly challenging in cases with complex molecular profiles, such as PIK3CA-positivity and PYGO2-negativity, as observed here. These alterations may influence tumor aggressiveness, therapeutic responsiveness, and clinical outcome, highlighting the need for a tailored management strategy. From a therapeutic standpoint, the activation of the PI3K/AKT/mTOR pathway represents a potential target for intervention. Several pharmacological agents aiming at this pathway have been explored in early-phase clinical trials. For example, alpelisib, a selective PI3Kα inhibitor, has shown activity in solid tumors harboring PIK3CA mutations. Although it is currently approved for certain breast cancers, its role in HCC is still under investigation. Similarly, everolimus, an mTOR inhibitor, has been evaluated in HCC settings but with modest success as monotherapy. Combination strategies involving PI3K/mTOR inhibitors and other agents are being explored to overcome resistance mechanisms and improve efficacy.

In the context of immunotherapy, HCC has become one of the few solid tumors where immune checkpoint inhibitors (ICIs) have been integrated into standard care. Atezolizumab (anti-PD-L1) combined with bevacizumab (anti-VEGF) has emerged as a preferred first-line regimen in patients with preserved liver function. This combination takes advantage of synergistic mechanisms: immune activation and angiogenesis inhibition. Other ICIs such as nivolumab and durvalumabare are being tested either alone or in combination with other systemic therapies in both frontline and second-line settings. However, it is unclear how molecular signatures such as PYGO2-negativity affect the tumor’s immunogenicity. Some studies suggest that low Wnt/β-catenin activity might be associated with a more favorable immune microenvironment, potentially increasing responsiveness to ICIs. Conversely, activation of the PI3K pathway has been linked in other cancers to immune exclusion, indicating a possible need for dual-targeting approaches (e.g., PI3K inhibitor + PD-1 blockade).

The challenge of therapeutic selection is compounded by limited access to molecular profiling in many centers, especially in routine clinical practice. Even when alterations are identified, the lack of approved targeted agents for HCC limits the immediate therapeutic value. Consequently, enrollment in molecularly stratified clinical trials becomes a crucial opportunity. Trials investigating novel agents that inhibit PI3K, AKT, or mTOR—either alone or in combination with ICIs—could offer potential benefit in such cases. Another avenue under investigation is the use of tumor-derived organoids or patient-derived xenografts (PDXs) for drug sensitivity testing. These platforms, though not yet standard in clinical care, may eventually guide treatment in difficult cases. Finally, given the rapid decline in liver function observed in this patient, the integration of supportive care measures remains paramount. These include management of complications such as ascites, hepatic encephalopathy, or coagulopathy, which not only impact quality of life but also determine eligibility for further systemic therapies.

It should be mentioned that this is a case report and the results obtained (PYGO2, PIK3CA) are limited to this strict case.

In summary, while no standard therapy currently exists for HCC with this precise molecular profile, a rational approach includes leveraging available systemic agents—particularly ICIs—and considering investigational therapies when feasible. Continued research and access to clinical trials are essential for improving outcomes in such biologically aggressive tumors.

## 4. Conclusions

In a case of hepatocellular carcinoma (HCC) which was PIK3CA-positive and PYGO2-negative, several key aspects of the tumor’s behavior, prognosis, and treatment options can be inferred. The PIK3CA mutation suggests activation of the PI3K/AKT/mTOR pathway, a critical signaling pathway involved in cell survival, proliferation, and metabolism. This mutation can lead to uncontrolled tumor growth and resistance to cell death mechanisms (apoptosis). Tumors that lack PYGO2 may not show the same types of tissue remodeling or regenerative features driven by the Wnt/β-catenin pathway, which could make the tumor behave differently from others that are Wnt-positive. PIK3CA-positive tumors are often associated with worse prognosis due to the aggressive nature of the PI3K/AKT pathway activation. This is linked to higher chances of metastasis, recurrence, and resistance to therapies that do not target this pathway. The negative PYGO2 status does not necessarily indicate poor prognosis directly, but it suggests that the tumor may not benefit from therapies targeting the Wnt/β-catenin pathway. It also implies that the tumor may not follow the common genetic patterns seen in other HCCs. The combination of immune checkpoint inhibitors (e.g., anti-PD-1/PD-L1 therapies like nivolumab or pembrolizumab) with targeted therapies may offer benefits, as the tumor could be more prone to immune evasion mechanisms due to its molecular profile. In this particular case of PIK3CA-positive and PYGO2-negative HCC, we should expect an aggressive tumor with PI3K pathway activation. Treatment will likely focus on targeting the PI3K/AKT/mTOR pathway, and the negative PYGO2 status suggests that Wnt/β-catenin-targeted therapies may not be useful. Due to the aggressive nature of PIK3CA mutations, close monitoring and consideration of immunotherapy and targeted treatments will be important.

Further studies are needed to understand the interrelationship between biomarkers and prognosis in these patients. Future work should also delineate PYGO2-linked microRNA networks in HCC to refine prognostic stratification and identify combinatorial targets.

## Figures and Tables

**Table 1:** Laboratory results recorded during the hospitalization time.

Test	Results	UM	Normal Values
White blood cells (WBC)	23.13	10^3^/μL	4.0–10.0
Neutrophils (NEU)	9.63	10^3^/μL	2.4–6.5
Lymphocytes (LYM)	5.62	10^3^/μL	1.0–4.0
Monocytes (Mono)	0.58	10^3^/μL	0.3–1.0
Red blood cells (RBC)	6.87	10^6^/μL	3.8–5.1
Hematocrit (HCT)	30.89	%	35–47
Platelets	80	10^9^/μL	150–400
Hemoglobin (HGB)	7.6	g/dL	13.2–17.3
MCV	82	FL	80–100FL
MCHC	40	g/dL	31–36
MCH	38	Pg	24–32
pH	7.23	-	7.35–7.45
Serum creatinine	1.5	mg/dL	0.10–1.2
Glycemia	102	mg/dL	65–115
Glomerular filtration rate (GFR)	60.26	mL/min/1.73 m^2^	>90 mL/min/1.73 m^2^
Uric acid	5.5	mg/dL	3.5–7.2
Aspartate aminotransferase (AST/GOT)	125	U/L	5–34
Alanine aminotransferase (AST/GOT)	180	U/L	0–55
Bilirubin	3.2	mg/dL	0.2–1.2
Cholesterol (CHOL)	189	mg/dL	0–199
HDL CHOL	55	mg/dL	40–60
LDL CHOL	98	mg/dL	<100
INR	3.01	-	0.8–1.2
PT	20	Seconds	10–13
APTT	52	Seconds	25–36
Fibrinogen	220	mg/dL	130–330
HBsAg	Negative		Negative
Anti-HBc IgM + IgG	Negative	-	Negative
Anti-HBs	14	mUi/mL	>10 mUi/mL
PCR for HBV-DNA	Negative	Copies/mL	Negative
Anti-HCV	Negative	-	Negative
HIV (Ab + Ag p24)	Negative	-	Negative
Serum albumin	2.2	g/dL	3.4–5.4
Lactate dehydrogenase	360	U/L	140–280
Alkaline Phosphatase	289	IU/L	44–147
Alpha 1 antitrypsin	378	mg/dL	80–220
Alpha fetoprotein	420	ng/dL	0–40
CRP	52	mg/dL	0–5.0
ESR	50	mm/h	2–30.0
Serum iron	48	Mcg/dL	60–160
Carcinoembryonic antigen (CEA)	3.1	Ng/mL	0–2.9
Carbohydrate antigen 19-9 (CA 19-9)	58	u/mL	0–37 u/mL

**Table 2:** PIK3CA mutations and cancer type associations.

Mutation Type	Description	Mutation Location	Mechanism of Action	Effect on PI3K Signaling	Functional Consequence	Cancer Types Associated	Prevalence in Cancer	Therapeutic Implications	Ref.
Hotspot Mutations (Gain-of-function)	Frequent mutations leading to enhanced kinase activity.	Exon 9 (E545K), Exon 20 (H1047R)	Alterations in the helicoidal (Exon 9) and kinase (Exon 20) domains, causing abnormal activation.	Constitutive activation of PI3K, driving tumorigenesis	Promotes cell survival, growth, and proliferation; resistance to apoptosis	Breast, Colon, Endometrial, Ovarian, Glioblastoma, and others	High in breast cancer, colon cancer, and more	Target for PI3K inhibitors, potential biomarker for therapy	[18]
Point Mutations (Missense)	Single base substitutions that lead to amino acid changes, often at hotspot sites.	Exon 9 (E542K, E545K), Exon 20 (H1047R, H1047L)	Missense mutations in critical regions of the gene, particularly affecting kinase and helical domains.	Increased PI3K kinase activity, activating downstream signaling	Altered protein function, leading to dysregulated growth and survival	Breast, Colon, Ovarian, Endometrial, and others	Common in breast, colon, and other epithelial cancers	Targetable with PI3K-specific inhibitors or combination therapies	[19]
In-frame Insertions and Deletions (Indels)	Small insertions or deletions (indels) within critical regions, especially in the helical or kinase domains.	Helical and kinase domains (Exons 9–20)	In-frame alterations in the sequence, leading to disruptions in protein folding and function.	Can lead to hyperactivation or dysfunction depending on the site and nature of the change	Leads to continuous activation of PI3K pathway or disrupts normal regulation	Breast, Endometrial, Gastrointestinal, and others	Less frequent than point mutations, but significant in some cancers	May be sensitive to PI3K inhibitors, depending on the mutation	[20]
Copy Number Variations (CNVs)	Gene amplifications or deletions, often leading to altered expression levels of PIK3CA.	Entire PIK3CA gene or flanking regions	Amplification of the PIK3CA gene leads to overexpression, causing constitutive activation.	Overexpression of PIK3CA, leading to sustained PI3K signaling	Increased cell proliferation and survival due to overexpression	Breast, Ovarian, Endometrial, and other cancers	Gene amplification detected in ~30% of breast cancers	Can be targeted by drugs that reduce gene expression or inhibit downstream signaling	[21]
Complex Mutations and Fusions	Chromosomal rearrangements or translocations affecting PIK3CA or regulatory regions.	Chromosomal translocations, structural variants	Gene rearrangements that result in altered expression or function of PIK3CA protein.	May lead to aberrant PI3K pathway activation or altered protein interactions	Can result in dysregulated cell growth or resistance to apoptosis	Rare, but can be seen in some aggressive cancers	Rare but aggressive cancer subtypes	Potential for targeted therapies based on the specific fusion partners	[22]
Loss-of-function Mutations (Rare)	Mutations leading to loss of function, such as nonsense, frameshift, or splice-site mutations.	Various regions of PIK3CA gene	Frameshift or nonsense mutations that create truncated, nonfunctional proteins.	Loss of PI3K signaling, possibly inhibiting cancer cell growth or promoting apoptosis	May have tumor-suppressive effects; reduced activation of the PI3K pathway	Rare, seen in some contexts like neurodevelopmental disorders or specific cancers	Very low prevalence in cancer, mostly in benign conditions	Potential for targeting with PI3K agonists to restore function	[23]

**Table 3:** PYGO2 mutations, molecular mechanism and examples of cancer type associations.

Mutation Type	Description	Molecular Mechanism	Functional Consequences	Cancer Pathology Implications	Examples of Related Cancer Types	Ref.
Point Mutations	Single nucleotide changes, including missense, nonsense, and silent mutations.	Missense: A single nucleotide change results in a different amino acid. Nonsense: Introduction of a premature stop codon, truncating the protein. Silent: A change in the codon, but no change in the amino acid.	Missense: Changes protein conformation, potentially impairing function or altering protein–protein interactions, destabilizing the protein. Nonsense: Leads to a truncated protein that lacks functional domains, disrupting signaling pathways. Silent: Often neutral, but may still affect mRNA splicing or stability.	Missensemutations could lead to Wnt/β-catenin pathway activation, promoting cell proliferation and survival. Nonsense mutations can result in the loss of tumor suppressor function by generating truncated, nonfunctional proteins, thereby facilitating uncontrolled cellular proliferation. Meanwhile, silent mutations, although not altering the amino acid sequence, may influence mRNA splicing or gene expression regulation, potentially exerting subtle but significant effects on tumorigenesis.	Colorectal Cancer: Missense mutations in PYGO2 could activate Wnt signaling, contributing to early tumor formation. Breast Cancer: Nonsense mutations leading to loss of function may increase cellular resistance to apoptosis. Lung Cancer: Altered Wnt signaling through PYGO2 mutations can drive tumor progression and metastasis.	[30]
Insertions/Deletions (Indels)	Insertion or deletion of nucleotides, causing reading frame shifts or amino acid changes.	Frameshift: Insertion/deletion of nucleotides not in multiples of three. In-frame Indels: Insertion/deletion of nucleotides in multiples of three, altering the protein sequence.	Frameshift: Alters the reading frame, creating a dysfunctional protein downstream. This can affect key signaling pathways like Wnt. In-frame Indels: Affects key amino acids, potentially altering protein function or disrupting its interaction with cofactors or other proteins.	Frameshift mutationscan lead to loss of function in tumor suppressor pathways (like Wnt signaling) or cause the gain of function in oncogenes, promoting tumorigenesis. In-frame Indelscould disrupt binding sites for cofactors in Wnt signaling, leading to enhanced cell proliferation.	Gastric Cancer: Frameshift mutations in PYGO2 could contribute to cancer progression by disabling tumor suppressor functions. Lung Cancer: In-frame indels may enhance Wnt/β-catenin pathway activity, leading to uncontrolled growth. Endometrial Cancer: Indels causing structural changes in PYGO2protein affect cell differentiation and proliferation.	[31]
Splice Site Mutations	Mutations in splice donor/acceptor sites, leading to incorrect mRNA splicing.	Disrupts the precise splicing process, leading to the inclusion of introns or the exclusion of exons.	- Results in alternative splicing, producing truncated proteins or dysfunctional protein isoforms. Mis-spliced mRNA may be degraded or lead to a protein that lacks key functional domains necessary for Wnt signaling.	Aberrant splicingcan produce proteins that either act as oncogenes (if gain-of-function) or tumor suppressors (if loss-of-function), promoting tumorigenesis. Mutations in PYGO2 could generate spliced isoforms that activate Wnt/β-catenin, driving cell survival and division.	Prostate Cancer: Aberrant splicing of PYGO2could lead to the production of a dominant-negative protein, contributing to resistance to apoptosis. Lung Cancer: Splice variants of PYGO2 may facilitate the tumorigenic effects of Wnt pathway dysregulation. Breast Cancer: Incorrect splicing may lead to loss of cell cycle regulation, contributing to tumor progression.	[32]
Copy Number Variations (CNVs)	Alterations in the number of gene copies, such as duplications or deletions	Duplication: Multiple copies of PYGO2 gene lead to overexpression. Deletion: Loss of one or more copies of PYGO2 gene, reducing expression	Duplication: Overexpression of PYGO2 leads to excessive activation of Wnt signaling, promoting cell proliferation and resistance to cell death. Deletion: Insufficient expression of PYGO2 disrupts Wnt signaling, potentially impairing developmental processes and tissue homeostasis	Gene duplicationcan enhance PYGO2expression, amplifying Wnt/β-catenin signaling, which promotes tumor initiation, progression, and metastasis. Gene deletionin PYGO2 can impair tumor suppressor functions, leading to genome instability and increased metastatic potential	Breast Cancer: Overexpression of PYGO2through gene duplication has been linked to enhanced metastatic behavior. Glioblastoma: Loss of PYGO2 function due to deletion has been associated with increased tumorigenicity and poor prognosis. Ovarian Cancer: Deletions of PYGO2 can cause dysregulated cell differentiation and growth.	[33]
Regulatory Region Mutations	Mutations in promoter, enhancer, or silencer regions affecting PYGO2 expression.	Promoter Mutations: Alteration of transcription factor binding sites. Enhancer/Silencer Mutations: Disruption of cis-regulatory elements.	Overexpression: Increased PYGO2 expression may hyperactivate Wnt signaling, leading to tumor initiation and progression.Underexpression: Reduced expression of PYGO2 results in loss of function, potentially leading to unchecked cellular proliferation.	Overexpression of PYGO2has been shown to activate the Wnt/β-cateninsignaling pathway, which plays a critical role in promoting cell cycle progression, cellular invasion, and metastatic dissemination. Conversely, underexpression of PYGO2 may result in impaired regulation of cellular differentiation, thereby facilitating malignant transformation.	Colon Cancer: Overexpression of PYGO2 through promoter mutations activates Wnt signaling and accelerates tumor progression.Hepatocellular Carcinoma: Disruption of regulatory regions leads to PYGO2 silencing, impairing cell differentiation and promoting carcinogenesis.Leukemia: Deregulated PYGO2 expression contributes to leukemogenesis by promoting uncontrolled cell proliferation.	[34]
Large-Scale Structural Variants	Chromosomal rearrangements, such as inversions, translocations, and deletions, affecting PYGO2.	Inversion: Reversal of a chromosomal region containing PYGO2.Translocation: Movement of PYGO2 to a new chromosomal location. Deletion: Loss of the region containing PYGO2.	Inversion: May disrupt PYGO2’s expression or function by altering its regulatory landscape.Translocation: PYGO2 may be placed under the control of an oncogenic promoter, driving overexpression. Deletion: Complete loss of PYGO2function, resulting in defective signaling and loss of tumor suppressor functions.	Inversions and translocationscould place PYGO2 under the control of strong oncogenic promoters or fuse it with other genes, driving tumorigenesis. Gene deletions result in the loss of Wnt signaling, increasing susceptibility to cancer progression and metastasis	Leukemia: Chromosomal translocations involving PYGO2 lead to fusion genes, driving leukemogenesis. Breast Cancer: Rearrangement or deletions of PYGO2 are linked to poor prognosis due to loss of cell cycle regulation. Ovarian Cancer: Structural variants of PYGO2 contribute to dysregulated cellular pathways, facilitating tumor growth and spread.	[35]
Epigenetic Modifications	DNA methylation, histone modification, and chromatin remodeling that regulate PYGO2 expression.	DNA Methylation: Addition of methyl groups to cytosine residues in the promoter region, silencing gene expression. Histone Modifications: Acetylation or methylation of histones that impact chromatin accessibility and gene expression.	Gene silencing: Methylation of PYGO2’s promoter can lead to loss of expression, disrupting the control of cell proliferation.Gene activation: Loss of methylation or histone acetylation can increase PYGO2 expression, pushing cells into unregulated proliferation.	DNA methylation silencingof PYGO2 may contribute to tumorigenesis by silencing a key tumor suppressor. Increased expressiondue to loss of silencing or aberrant histone modification enhances tumor cell survival and metastasis.	Colon Cancer: DNA methylation of PYGO2is linked to silencing in tumor cells, allowing cancer progression. Lung Cancer: Epigenetic silencing of PYGO2disrupts normal cellular homeostasis and promotes carcinogenesis. Hepatocellular Carcinoma: Histone modifications leading to overexpression of PYGO2 contribute to aggressive tumor behavior.	[36]

## Data Availability

The original contributions presented in this study are included in the article. Further inquiries can be directed to the first author.
